# TZM-gfp cells: a tractable fluorescent tool for analysis of rare and early HIV-1 infection

**DOI:** 10.1038/s41598-020-76422-6

**Published:** 2020-11-16

**Authors:** David W. Gludish, Saikat Boliar, Shannon Caldwell, Dumizulu L. Tembo, Elizabeth T. Chimbayo, Kondwani C. Jambo, Henry C. Mwandumba, David G. Russell

**Affiliations:** 1 Department of Microbiology and Immunology, College of Veterinary Medicine, Cornell University, Ithaca, NY 14853 USA; 2 Malawi-Liverpool-Wellcome Trust Clinical Research Programme, University of Malawi College of Medicine, Blantyre, Malawi; 3 Department of Clinical Sciences, Liverpool School of Tropical Medicine, Liverpool, UK

**Keywords:** HIV infections, Retrovirus, HIV infections

## Abstract

Here we describe TZM-gfp, a novel HIV-1 reporter cell derived from the same parental clone JC.53, used previously to generate the widely-utilized indicator cell line TZM-bl. We re-engineered JC.53 cells to express GFP under regulation of HIV Tat and Rev. We characterize the new reporter cell line to show that TZM-gfp cells are equally susceptible to HIV infection, exhibit minimal background signal, and can report HIV infection in rare cells from a bulk population of experimentally-infected human monocyte-derived macrophages. We demonstrate the utility and sensitivity of the cells in detection of even a single HIV-positive macrophage by fluorescence-assisted correlative electron microscopy, using the GFP signal to guide imaging of HIV virions in primary co-culture. Finally, we used TZM-gfp cells for viral capture during co-culture with human peripheral blood mononuclear cells, showing that TZM-gfp can support outgrowth and analyses of patient-derived primary HIV-1 isolates.

## Introduction

The global effort to study and therapeutically target HIV replication has been greatly advanced by the wide adoption of defined cell lines for in vitro study and quantitation of viral isolates. While many of these reagents are human lymphoblastic leukemia cell lines that grow in suspension culture, a notable exception is the HeLa-derived HIV-1 indicator cell platform TZM-bl, which are adherent, non-immunological cells engineered to overexpress the HIV-1 co-receptors CD4, CCR5, and CXCR4^1^. TZM-bl cells feature an HIV Tat-responsive long terminal repeat (LTR) promoter driving the expression of beta-galactosidase and firefly luciferase, and have become a key reagent for the study of basic HIV biology, drug screens, and more recently in clinical studies of HIV persistence^2^.

Though TZM-bl and other cell lines are available to quantify outgrowth from human patient samples using robust enzymatic readout, investigators face a shortage of tools to probe HIV biology at the level of the individual cell. Given their wide adoption, we reasoned that TZM platform-based cells would be ideally suited to such studies, if re-engineered to generate a fluorescent signal upon HIV infection. We therefore developed TZM-gfp, an HIV indicator cell line derived from the JC.53 HeLa clone that is dependent on both Tat and Rev for expression of the *Renilla* GFP gene. We show that TZM-gfp can reliably report HIV replication following infection with cell-free viral stocks or during co-culture with infected human primary macrophages. We also demonstrate that these cells can be used in conjunction with correlative electron microscopy to detect and visualize virion production at the cellular level. Moreover, we show that TZM-gfp cells are able to capture and expand primary HIV-1 isolates from the peripheral blood mononuclear cells (PBMCs) from an infected donor. TZM-gfp cells afford new opportunities to study HIV infections using cell-based fluorescence, and are amenable to study infections by both cell-free virus such as from plasma and in a co-culture system with primary patient-derived cells. We envision TZM-gfp cells as an additional reagent that builds on the utility of the TZM-bl platform to study HIV infection at the population, cellular and ultrastructural levels.

## Results

### Generation of TZM-gfp cells for fluorescence readout during standardized HIV-1 infectivity assays

JC.53 cells are HeLa cell derivatives overexpressing the three major HIV-1 co-receptors, CD4, CCR5 and CXCR4, and were first described by David Kabat and colleagues^1^. Subsequent work in the lab of John Kappes produced JC.53-bl cells (later renamed TZM-bl), which added *E. coli* beta-galactosidase (β-gal) and firefly luciferase reporter genes to quantify HIV replication or inoculum infectivity^3^. Providing sensitive enzymatic readout, the TZM-bl cell line has been almost universally adopted in HIV basic research, and has more recently been utilized as a viral outgrowth assay platform for clinical samples^2^.

**Figure 1:**
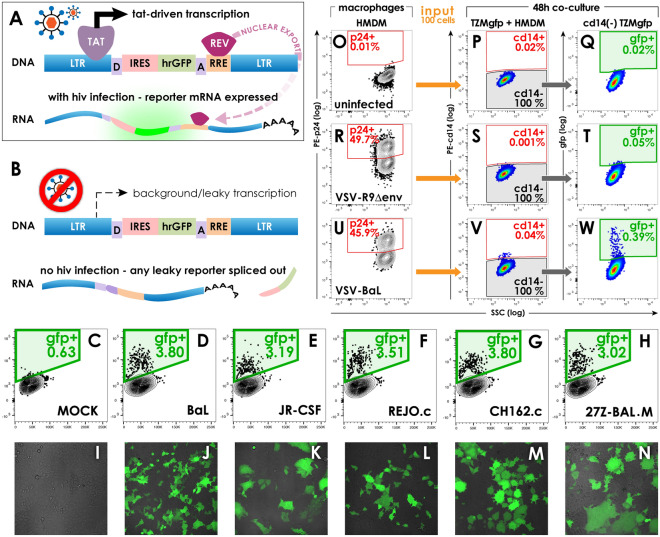
TZM-gfp cells produce hrGFP reporter signal upon active infection with diverse HIV strains.** A** Schematic of Tat- and Rev-responsive lentiviral reporter construct p-NL-GFP-RRE(SA) during HIV infection. Tat drives strong transcription at the HIV-1 LTR, while Rev protein binds the RRE on transcribed mRNA for rapid nuclear export, subverting nuclear splicing machinery. **B** In the absence of HIV infection, any background transcripts are retained in the nucleus in the absence of Rev protein, leading to splicing out of hrGFP reporter, and no fluorescence signal. **C–N** Direct infection of TZM-gfp with a panel of cell-free viral stocks. Compared to mock-infected cells (**C,I**), all infected wells develop GFP reporter signal by 48 h post-infection; flow cytometry dot plots for GFP (y-axis) versus forward scatter (x-axis) are shown. The percentage of GFP-positive cells versus the bulk TZM-gfp population is indicated inside each green gate, the strain of HIV is provided in black text for each dot plot (**C**–**H**) and its corresponding confocal fluorescence micrograph (**I**–**N**) in the row below. **O–W** Co-culture of HMDM with TZM-gfp to report GFP induction after mock-infection (**O,P,Q**), single-round VSV-G-R9Δ*env* infection (**R,S,T**), or replicating double-envelope infection with VSV-G-BaL (**U,V,W**). **O,R,U** HMDM were harvested, fixed and stained to analyze HIV penetrance by p24 antigen staining (KC57-RD-1, Beckman Coulter) compared to uninfected control macrophages, **O**. One hundred live cells from the cultures stained in O,R,U were added to established TZM-gfp monolayers and cultured for 48 h. Cultures were harvested by gentle trypsin treatment, gated on CD14-negative TZM-gfp cells (**P,S,V**) and analyzed for GFP expression (**Q,T,W**) by flow cytometry. The HIV infection inoculum is indicated inside the p24 dot plots (**O,R,U**), and the percentage of gated cells is indicated inside each gate.

To produce a fluorescent HIV indicator cell line, we selected pNL-GFP-RRE(SA)^4^ (Fig. 1A), a non-self-inactivating lentiviral reporter that harbors the HIV-1 LTR and HIV-1 major donor and acceptor splice sites flanking the reporter gene hrGFP (human-optimized Renilla GFP). During HIV infection, Tat drives transcription of the reporter mRNA, on which the Rev responsive element (RRE) is bound by HIV Rev protein for rapid nuclear export and subversion of the nuclear spliceosome. Without HIV infection or Tat protein, the LTR is transcriptionally inactive. Similarly, in the absence of HIV Rev protein, any ‘leaky’ transcripts are processed in the nucleus, where the hrGFP coding sequence is spliced out of the nascent mRNA between the HIV major donor and acceptor splice sites (Fig. 1B). Together these features ensure that presence of both Tat and Rev proteins regulate clean, robust reporter induction.

JC.53 cells were transduced with pNL-GFP-RRE (SA) and cloned by limiting dilution in 96-well plates. Expanded subclones were screened for reporter readout following infection with HIV-1 ADA^5,6^, both by direct infection with cell-free HIV stocks and by co-culture with human monocyte-derived macrophages (HMDM) infected 14 days prior with vesicular stomatitis virus g-glycoprotein (VSV-G) pseudotyped ADA (data not shown). A single subclone (2H) was selected for its kinetics and strength of reporter induction (data not shown), and was renamed “TZM-gfp” for subsequent analyses.

To assess the susceptibility of TZM-gfp to diverse lab-adapted, patient-derived, or transmitted/founder HIV strains, cultures were infected with a panel of cell-free HIV stocks and live, unfixed cultures were analyzed by flow cytometry at 48 h (Fig. 1C–H), and by confocal microscopy at 72 h post-infection (Fig. 1I–N) under Biosafety Level 3 (BSL3) containment. GFP reporter signal developed in all infected cultures by 48 h, an early timepoint for TZM-gfp readout with cell-free stocks. TZM-gfp cells were similarly susceptible to infection with lab-adapted (BaL, Fig. 1D,J; JR-CSF, Fig. 1E,K), transmitted/founder clones (Subtype B/REJO.c, Fig. 1FL; Subtype C/CH162.c, Fig. 1GM), and a patient-derived, uncharacterized viral swarm (Subtype C/27Z-BAL.M, Fig. 1HN). We noted differences in the kinetics and/or extent of multinucleate syncytium formation among the HIV strains, an important strength of fluorescent reporters during infection assays (Fig. 1JL vs. KN).

### TZM-gfp reporter activity requires replicating HIV-1 infection

To demonstrate specificity of the hrGFP reporter readout, TZM-gfp cells were co-cultured with HMDM infected 3 days prior with laboratory strains of HIV-1. Macrophage infections were verified by flow cytometry at the time of harvest following p24-staining (Fig. 1ORU). One hundred harvested HMDM were added to established cultures of TZM-gfp cells, and co-cultures were incubated for 48 h. Trypsin-harvested co-cultures were strained (70 µM) for size selection, and gated on CD14 expression to (Fig. 1PSV) enrich for TZM-gfp cells in the analysis of GFP fluorescence. Control wells with uninfected (mock) HMDM exhibited no background GFP signal (Fig. 1Q, 0.02%) in the CD14^-^ (TZM-gfp) gate. Only in wells containing HMDM infected with replication competent HIV was robust GFP fluorescence observed in co-cultured TZM-gfp cells (Fig. 1W). To confirm that productive infection of TZM-gfp was necessary for reporter readout, separate wells of HMDM were infected with single round VSV-G-R9Δ*env* virions unable to generate a spreading infection, and used as input for TZM-gfp co-culture (Fig. 1R). CD14^-^ TZM-gfp cells from VSV-G R9Δ*env*-infected HMDM co-cultures (Fig. 1S) failed to generate GFP reporter signal (Fig. 1T, 0.05%). We confirmed that VSV-G-R9Δ*env* virus can drive robust reporter expression in TZM-gfp cells through direct infection with cell-free virus (data not shown). Together these data suggest that soluble Tat, and/or other mediators shed from cells harboring defective virus are insufficient to drive reporter expression in bystander TZM-gfp cells, and that GFP expression is only observed upon direct infection with replication-competent HIV.

### Extracellular Tat is insufficient for TZM-gfp reporter induction

**Figure 2:**
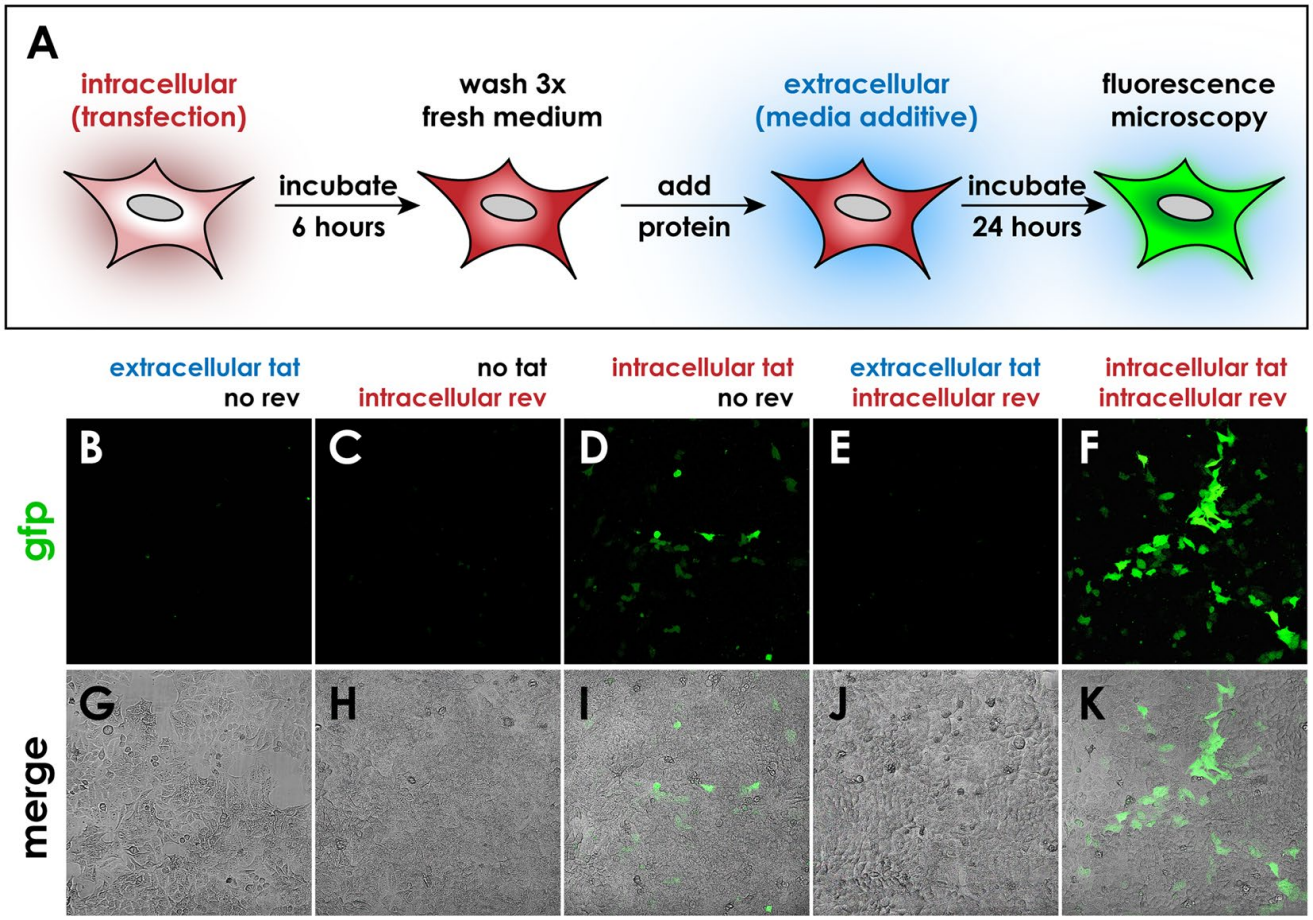
Extracellular Tat protein cannot induce reporter activity in TZM-gfp cells. **A** Experimental schematic showing steps of intracellular transfection followed by washing and addition of extracellular protein. Confocal fluorescence (**B–F**) and brightfield merged micrographs (**G–I**) of cells imaged 24 h after addition of extracellular protein. Cells treated either with extracellular Tat alone (**B,G**), intracellular transfected Rev alone (**C,H**), or with extracellular Tat together with intracellular transfected Rev (**E,J**) showed no GFP reporter induction 24 h after treatment. Mild GFP induction is seen after intracellular transfection of Tat (**D**,**I**), while a much larger increase in induction is observed with intracellular co-transfection of both Tat and Rev (**F**,**K**).

The requirement for intracellular Tat and Rev proteins during TZM-gfp reporter induction is an important criterion defining the stringency of an HIV indicator cell line. To probe this further, recombinant bioactive proteins were either transfected intracellularly alone or in combination, or used as medium additives; GFP fluorescence was monitored by live cell microscopy to assess reporter induction 24 h later (Fig. 2A). In single-protein treatments, extracellular Tat alone (300 ng/mL, Fig. 2BG) or transfected Rev alone (Fig. 2C,H) were insufficient to drive detectable accumulation of hrGFP. Importantly, extracellular Tat was unable to drive GFP reporter induction in Rev-transfected cells (Fig. 2EJ). By contrast, some baseline reporter induction was observed in a minority of cells transfected with intracellular Tat protein (Fig. 2D,I), while more robust and widespread induction was observed with intracellular co-transfection of both Tat and Rev protein (Fig. 2FK). These data reveal that TZM-gfp cells are insensitive to exogenous extracellular Tat (Fig. 2) or as bystanders during co-culture with HMDM bearing replication-defective HIV (Fig. 1T).

### TZM-gfp infection in co-culture is focal and cell-density dependent

**Figure 3:**
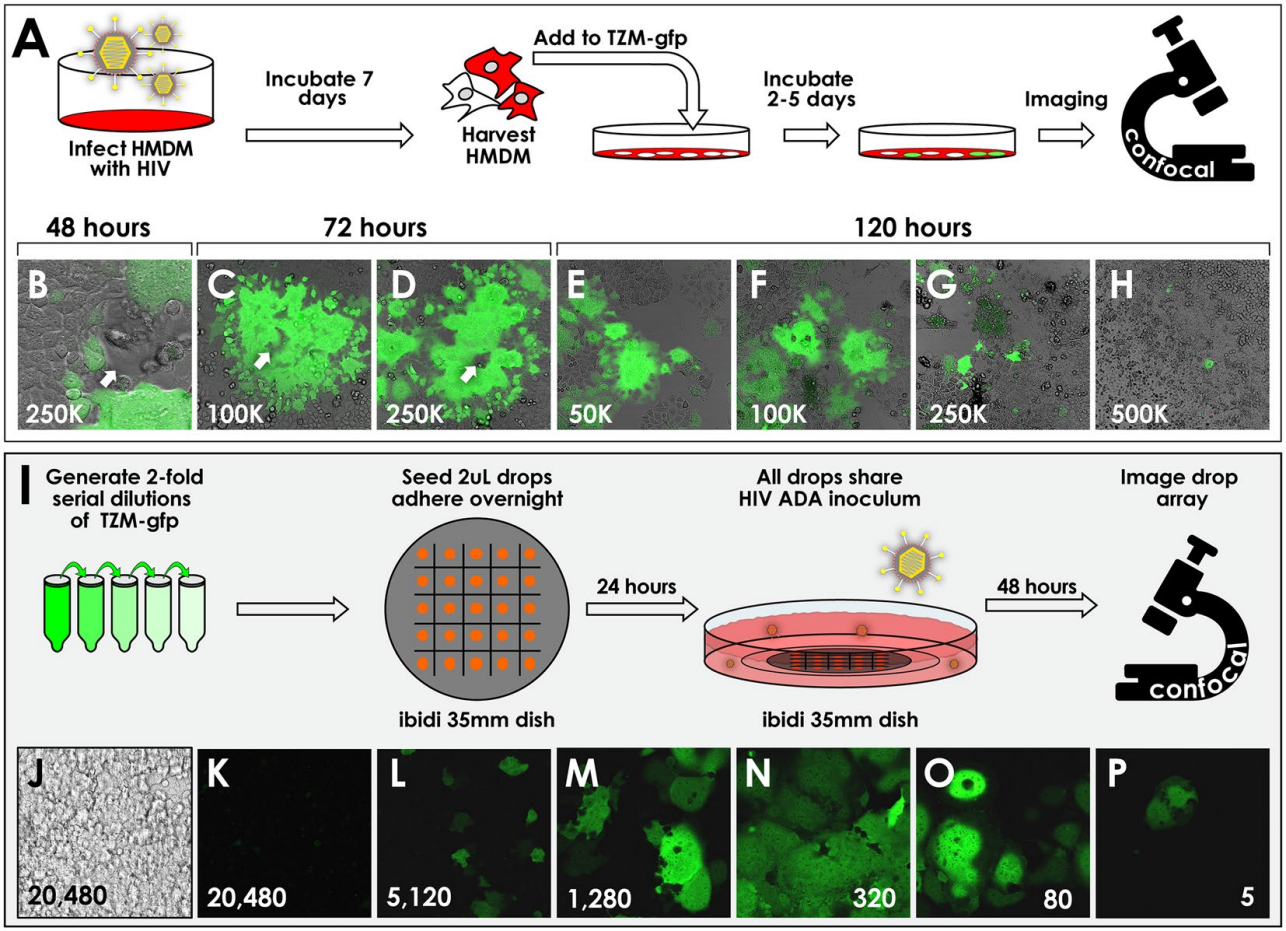
TZM-gfp cells are refractory to HIV-1 infection at high cell density, and report HIV infection even at clonal density.** A–H** HMDM co-culture with TZM-gfp at varying cell densities**. A** Experimental setup showing infection of HMDM in Teflon jars for convenient harvest at 14 days post-infection. Harvested HMDM are added onto monolayers of TZM-gfp cells seeded at four densities (**B–H**), showing inhibition at high density (**H**). Number of seeded TZM-gfp cells is indicated in each panel. **B** is shown at ×400 magnification to more clearly highlight typical macrophage morphology (white arrow) Initiating macrophages at various foci are indicated by white arrows. Other panels **C–H** are ×200 magnification. **I–P** Cell-free viral infection of serial dilutions of TZM-gfp cells in a drop culture assay.** I** Serial dilutions of TZM-gfp were plated in 2uL drops on a 5 × 5 array in ibidi 35 mm plates, and cells allowed to adhere overnight. Drop cultures were then overlaid with HIV-1 ADA supernatant such that all foci shared the same concentration of virus. Forty-eight hours post-infection, foci were examined for HIV infection by confocal micrsocopy (**J–P**). **J** Brightfield image of highest TZM-gfp density, 20,480 cells per drop. **K-P** Fluorescence micrographs of drop foci containing the indicated number of TZM-gfp cells plated.

TZM-gfp cells were envisioned as a tool to study qualitative phenotypes associated with rare or early HIV infection from laboratory or clinical samples of HIV. Limiting numbers of primary cells are often a hallmark of clinical samples, and maximizing reporter readout is important when HIV-infected cells are expected to be rare, such as from potential reservoir tissues or compartments. We therefore titrated TZM-gfp cell density to better characterize reporter performance with a fixed, low number of input primary cells. HMDM cultures infected 7 days prior were harvested and 100 cells were seeded on top of established TZM-gfp monolayers of differing densities (5 × 10^3^–1 × 10^5^ TZM-gfp cells/cm^2^), Fig. 3A.

At early timepoints during co-culture (48-72 h), foci of TZM-gfp reporter activity were observed in regions close to adhered macrophages (Fig. 3B–D), often with formation of small multinucleate syncytia. By 120 h TZM-gfp reporter activity was detectable within discrete TZM-gfp cells in areas lacking adhered macrophages (Fig. 3E–H). These observations suggest that cell-to-cell spread is the primary means of viral spread under these conditions.

HIV infection of TZM-gfp cells also appeared dependent on their seeding density. At lower densities (50,000 and 100,000 cells per 35 mm dish), robust GFP signal developed at multiple foci across the culture by 48 h (Fig. 3B–G). Significantly less infection was observed at higher density (500,000 cells per dish, Fig. 3H), despite equal loading of 100 HMDMs across all TZM-gfp densities. These data are consistent with rigorous, quantitative studies published on the related TZM-bl cell line, where high cell density is inversely correlated with HIV infection outcome^7^.

To determine whether increasing cell density of TZM-gfp similarly interfered with susceptibility to infection by cell-free HIV viral stocks, we seeded serial twofold dilutions of TZM-gfp cells in a 5 × 5 matrix of 2 μL drops in coverslip dishes (ibidi), allowed them to adhere, and challenged the whole dish with HIV-1 ADA virions (Fig. 3I). With increasing cell density, a dose-dependent decrease in infection outcome was observed at the center of each cell focus (Fig. 3J–P). These more qualitative results are comparable with existing quantitative data in TZM-bl cells^7^, and suggest that high density inhibition of HIV infection is a common feature of TZM-bl and TZM-gfp during both fluid-phase viral infection and cell-to-cell HIV transfer in co-culture.

### Correlative electron microscopy of HIV-positive foci in TZM-gfp

**Figure 4:**
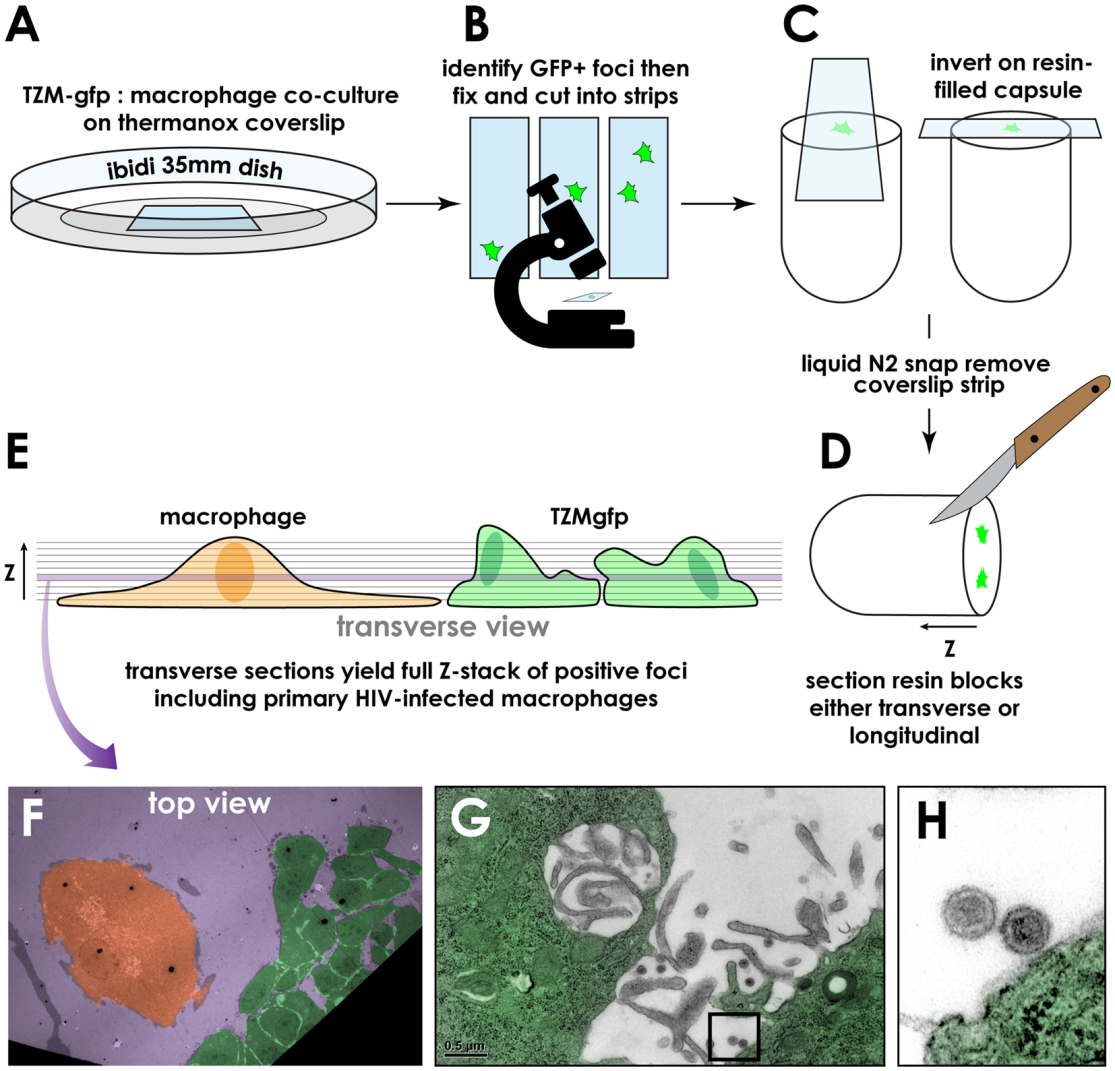
GFP-guided correlative electron microscopy identifies HIV virions in and around HMDM-associated foci. **A–E** TZM-gfp were co-cultured with 100 HIV-infected HMDM. **B** Two days after plating, cultures were examined for reporter activity and regions selected for embedding. **C** Strips of coverslip were cut and embedded on top of a resin-filled Beem capsule, and snapped off under liquid nitrogen. **D,E** Sections were cut for electron microscopy (EM) analyses. **F** Pseudocolored top view of the cellular structures visible from the purple section in panel **E**. **G,H** Infected TZM-gfp cells (green pseudocolor) show virions associated with, or in close proximity to the cell surface. **I** higher magnification of indicated region in panel **H**.

Very little is known about HIV genotypes that replicate in vivo but show limited fitness for outgrowth in vitro. To learn whether rare GFP-positive foci in TZM-gfp co-cultures could be evaluated for productive HIV infection at very early timepoints, we turned to correlative electron microscopy (EM). HIV-infected HMDM were seeded onto TZM-gfp monolayers on Thermanox coverslips (Nunc) cultured in 6-well dishes. A schematic of this experiment is shown in Fig. 4. At 72 h of co-culture, GFP-positive foci were located by fluorescence microscopy (Fig. 4A,B), then coverslips were fixed and cut into strips (Fig. 4B) for staining and embedding on top of a Beem capsule (Fig. 4C). Following polymerization, the strip was snapped off under liquid nitrogen (Fig. 4D), and sectioned from the apical side down (Fig. 4E). Electron micrographs revealed HIV infection in TZM-gfp cells near adhered HMDM (Fig. 4FG), including budding of new HIV from the cell surface (magnified in Fig. 4H), permitting detection of virion production early during ex vivo HIV outgrowth.

### TZM-gfp cells capture and replicate primary HIV-1 isolates.

**Figure 5:**
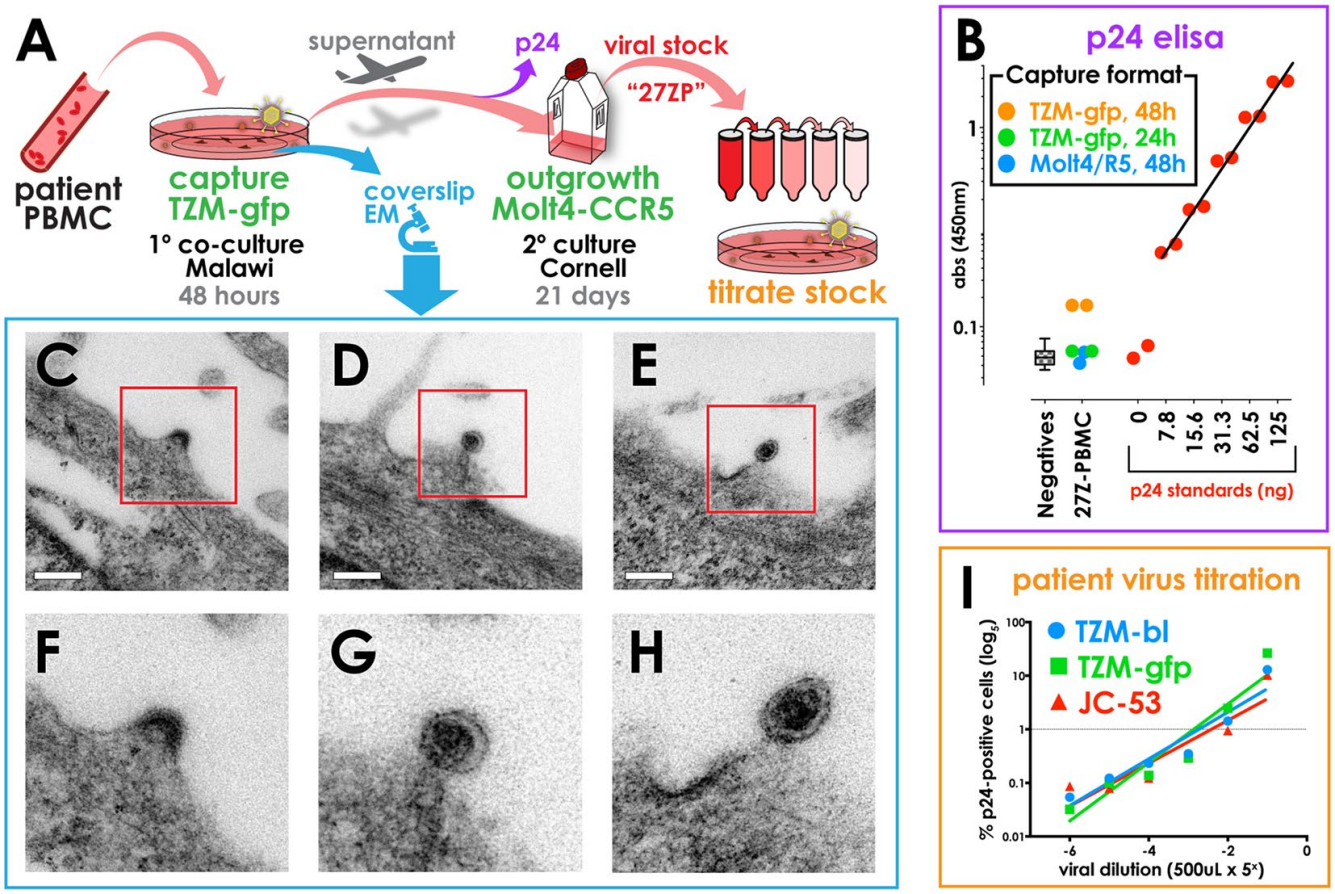
Replication of a primary human HIV-1 isolate in TZM-gfp cells.** A** Schematic of primary co-culture for viral capture, and subsequent processing of patient-derived supernatants. **B** p24 ELISA of primary co-culture supernatants harvested at the purple arrow in panel **A**. Pooled samples negative for p24 (hatched box) and a standard titration curve (red data points) demonstrate background signal and detection limits. Only PBMC:TZM-gfp co-culture supernatants from HIV-infected participant 27Z-PBMC exhibit p24 signal above background levels by 48 h of culture (orange); however p24 concentrations cannot be reliably quantified at this early timepoint, and do not score above the lowest standard (red data points in **B**). **C–H** Despite unreliable p24 levels, transmission electron microscopy of TZM-gfp co-culture coverslips corresponding to the orange data points in panel **B** reveal many lentiviral particles budding from the surface of TZM-gfp cells. Red outlined regions in **C**,**D**,**E** (scale bars = 200 nm) are magnified in **F**,**G**,**H** respectively. This visual confirmation of HIV virion production in primary co-culture was validated through generation of high-titer virus stocks “27ZP” following 21 days of secondary outgrowth in Molt4-CCR5 cells. **I** “27ZP” stock infectivity was titrated in TZM-bl (blue), TZM-gfp (green) and JC.53 cells (red), and the fraction of p24-positive cells at each dilution was determined by flow cytometry 36 h post-infection. Trendlines in each corresponding color reveal comparable susceptibility of these three cell lines to infection with primary, patient-derived HIV inoculum.

TZM-bl cells have been used to capture primary HIV-1 isolates, so we sought to determine if TZM-gfp could capture primary HIV-1 directly from donor-derived samples. We plated adherent TZM-gfp cells on coverslips for co-culture with PBMCs from an HIV-1-infected antiretroviral therapy (ART)-naïve Malawian adult volunteer, whose plasma HIV viral load was 1.1 × 10^4^ copies/mL (Fig. 5A). While no p24 gag antigen was detected after 24 h of PBMC:TZM-gfp co-culture (Fig. 5B, green data points), a small but detectable increase in supernatant p24 was seen by 48 h in these cultures (Fig. 5B, orange data points). By contrast to TZM-gfp co-cultures at 48 h, equal numbers of PBMC from this patient co-cultured with Molt4-CCR5 cells had no detectable p24 antigen at the same timepoint (Fig. 5B, blue data points), arguing that increases in p24 at 48 h of TZM co-culture was due to transfer and productive infection of TZM-gfp cells, and not sustained release of virions from infected primary PBMC. Together, our findings suggest increased cell-to-cell transfer kinetics in TZM-gfp cells, supporting more rapid p24 production than in lymphoid Molt4-CCR5 cells at these early timepoints.

Co-culture supernatants with p24 antigen levels that increase over time must contain productive HIV infection in patient/primary cells, neighboring reporter cells, or both. To confirm the presence of HIV virions, coverslips from patient-derived co-cultures were fixed and processed for transmission electron microscopy. Indeed, we confirmed that TZM-gfp cells in PBMC co-culture harbored lentiviral virions at 48 h (Fig. 5C–H). Following subsequent longer-term outgrowth culture in SupT1-CCR5 cells, the supernatant from these primary TZM-gfp co-cultures produced high-titer viral stock, designated “27ZP” (Fig. 5A,I).

### Comparative analysis of TZM-platform cell lines

**Figure 6:**
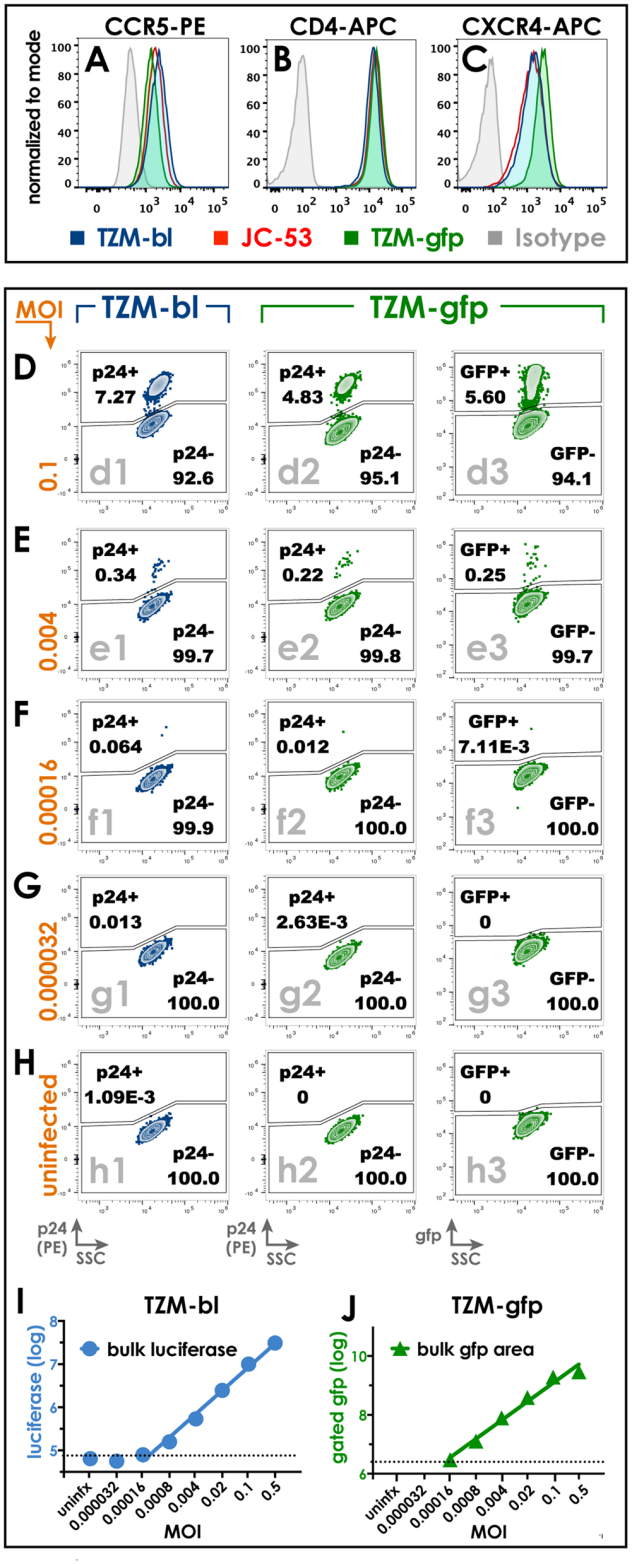
Comparative susceptibility of TZM-bl, TZM-gfp and JC.53 cells to HIV-1 infection.** A–C** Cell surface staining of three cell lines with molecular determinants of HIV-1 infectivity: (**A**) CCR5, (**B**) CD4, and (**C**) CXCR4. **D–H** Flow cytometric dot plots of a serial fivefold dilution series of HIV-1 BaL (starting at MOI 0.5), at 48-h post-infection**.** The multiplicity of infection (MOI) is indicated on the left side of each row in orange; some dilutions are not shown. **D–H.1,2 (Column 1 and 2)** show p24 positive cells from the singlet gate of TZM-bl (Column 1) and TZM-gfp (Column 2). **D–H.3 (Column 3)** shows GFP-positive cells from the singlet gate of TZM-gfp cells. In all plots, the fraction of gated cells is indicated in boldface type below the population name within each gate. **I** Bulk luciferase assay output is plotted as luminescence for each of the dilutions corresponding to replicate wells from **D–H.1**. Cultures with MOI of 0.000032 are indistinguishable from uninfected wells. **J** Bulk GFP fluorescence (mean fluorescence intensity of GFP^+^ gate *x* number of gated cells) is plotted for each dilution in **D–H.3**. No GFP^+^ cells were gated in MOI 0.000032 and uninfected wells.

We next compared directly the performance of TZM-platform cells with patient-derived virus. Serial five-fold dilutions of isolate 27ZP, derived from a Malawian adult volunteer (Fig. 5A–H), were used to inoculate TZM-gfp, TZM-bl and JC.53 cells. At 36 h post-infection, cells were harvested for intracellular p24 staining and flow cytometric analyses (Fig. 5I). The fraction of p24-positive gated cells was plotted for each viral dilution, revealing comparable infection penetrance in JC.53, TZM-bl and TZM-gfp cells exposed to equivalent amounts of virus. Perhaps unsurprisingly, these performance data were underscored by nearly equal surface expression of HIV receptors CCR5 (Fig. 6A), CD4 (Fig. 6B), and CXCR4 (Fig. 6C) in JC.53 cells and their derivatives TZM-bl and TZM-gfp cells.

TZM-gfp cells were designed as a companion (not replacement) reagent for TZM-bl, thus we compared susceptibility to infection and reporter output between these cells as a reference point in a fivefold dilution series of HIV-1 BaL (Fig. 6D–J). Infection penetrance monitored by flow cytometry using p24 staining (Fig. 6D–H, columns 1, 2) revealed that TZM-bl and TZM-gfp cells had highly similar susceptibility profiles. Furthermore, strong correlation of p24 penetrance (Fig. 6D–H, column 2) was observed with GFP-positive fractions in TZM-gfp cells (Fig. 6D–H, column 3). While the limit of detection in these experiments using p24 flow cytometry detection was an MOI of 0.000032, reporter induction for TZM-bl or TZM-gfp was not detected below MOI 0.00016 (GFP flow cytometry, Fig. 6FG.3,J; Luciferase enzyme assay, Fig. 6I). Reporter induction above background (mean fluorescence intensity [MFI] of GFP^+^/GFP^−^ cells) was consistent in TZM-gfp cells at 17–19-fold, even in wells with very few GFP-gated cells (Fig. 6F.3), showing nearly binary GFP reporter induction at 48-h post-infection. Indeed, when plotted as bulk GFP fluorescence (MFI × # of GFP-gated cells), TZM-gfp reporter activity could identify HIV-positive wells at the same sensitivity on a per-well basis as bulk luciferase assay in TZM-bl, despite the much higher fold-induction of luciferase (up to 1700-fold, Fig. 6I).

## Discussion

TZM-gfp cell offers a highly-specific, sensitive, readout for viral infection with single cell resolution. We believe that such an adherent fluorescent reporter cell platform offers certain advantages for studying single (vs. bulk) or early infection events from primary tissue, blood samples, or reservoir populations where the frequency of viral infection is known to be low. Recent quantitative viral outgrowth assay (QVOA)^8^ results have suggested that the primary QVOA assay underestimates quantitation of HIV reservoirs^2,9–11^. In this context, TZM-gfp cells represent an additional reagent capable of detecting HIV infection as fluorescent foci, thus facilitating study of early HIV isolates at cellular resolution without molecular cloning. Importantly, TZM-gfp cells are intended to explore qualitative, cell-level phenotypes that can augment the quantitative analyses that are more conveniently achieved with enzymatic reporters, such as with TZM-bl.

We show here that TZM-gfp performs comparably with other TZM-platform cells TZM-bl and JC.53. These characteristics are driven by near-identical cell surface expression of the HIV receptor machinery on these derivative cells, and are underscored by similar limitations for their use in culture. As previously reported, we confirm that TZM-bl and TZM-gfp cells exhibit strict density-inhibition of HIV susceptibility, arguing that overseeding or long-duration culture will rapidly inhibit HIV outgrowth within TZM-platform cell cultures. Diverse investigative approaches will leverage the strengths of either (or both of) enzymatic or fluorescence readout, but signal-to-noise ratio is a unifying limitation of both methods. Luciferase fold-induction was up to 1700-fold in TZM-bl, ascribed to the nearly absent background signal in TZM-bl. As expected from a fluorescent reporter, fold-induction was comparatively much weaker in TZM-gfp (19-fold in TZM-gfp; Fig. 6). Like any bulk analysis, this very strong luciferase induction in TZM-bl assays is limited by averaging over the entire lysate volume. By contrast, what TZM-gfp cells lack in signal-to-noise ratio, they also gain in specificity of a cell-level readout; in our hands, this compensated sufficiently to provide equivalent per-well sensitivity of luciferase and GFP in parallel cultures or TZM-bl and TZM-gfp, respectively. Our findings provide investigators an additional tool to confidently deploy in existing TZM-platform pipelines where cell-level or live-cell HIV reporting is of value.

TZM-gfp cells are particularly well suited to a co-culture system with the possibility of time lapse fluorescence imaging in live cells to study longitudinal expansion of early infection events. Such studies can focus on the behavior or phenotype of input primary cells as they seed infection in co-culture. Although we have presented data largely using macrophage co-culture assays, we also demonstrate the capture of HIV from patient-derived PBMCs to show that TZM-gfp cells support capture and outgrowth of primary HIV isolates from multiple cellular sources.

The specificity of the TZM-gfp reporter is governed by intracellular activity of both HIV-1 Tat and Rev proteins to drive GFP expression, representing a high level of stringency. We were surprised to learn that mild GFP expression could be elicited in TZM-gfp cells singly transfected with intracellular Tat, suggesting that in the context of strong Tat-driven transcription, some full length GFP mRNA was able to escape nuclear splicing in the absence of Rev. Thus, in TZM-gfp cells, spliceosome regulation of GFP reporter activity is not absolute. We confirmed, however, that extracellular Tat (a cell-permeant peptide) did not induce TZM-gfp reporter expression; this was true either as a high-concentration soluble culture medium additive in the presence of transfected Rev protein, or as released from HIV(Δenv)-infected HMDM in co-culture. We conclude that the two-factor (Tat, Rev) regulation of GFP reporter induction in TZM-gfp cells is sufficient to provide the sensitive and specific reporter induction so critical for confident detection of rare infection events. Subsequent verification that single, TZM-gfp fluorescent events in culture reflect productive HIV infections was achieved by correlative electron microscopy to image the production and release of HIV virions in situ. This demonstrates productive transfer of HIV infection and bypasses selection pressures or fitness requirements of prolonged in vitro outgrowth assays. Furthermore, the TZM-gfp culture system permits live monitoring of infection process over time, and the flexibility of longitudinal supernatant sampling for parallel molecular analyses.

In summary, we present TZM-gfp cells as an additional tool for HIV detection and characterization of infectivity in real time, and with single cell resolution. The capacity of TZM-gfp cells to report rare HIV infection events at the single cell level expands the options open to investigators interested in viral infectivity and outgrowth from primary sources.

## Methods

### Cells

TZM-bl cells were obtained through NIH AIDS Reagent Program, Division of AIDS, NIAID, NIH: TZM-bl cells (Cat#8129) from Dr. John C. Kappes, and Dr. Xiaoyun Wu. The parental JC.53 cells were a generous gift from Dr. David Kabat, OHSU. Affinofile-GGR cells were graciously provided by Dr. Benhur Lee, Mount Sinai. 293FT cells were purchased from Invitrogen, and human peripheral blood monocytes were purchased commercially from the University of Nebraska Medical Center Elutriation Core Facility.

### Cell culture

Human monocytes (University of Nebraska Medical Center Elutriation Core) were differentiated in 4 mL HMDM medium comprising high glucose DMEM medium with 1 mg/mL l-glutamine, 1 mM sodium pyruvate, 10 mM HEPES, 1 × Pen/Strep, and 10% pooled human AB-type serum (SeraCare) in Teflon (PTFE) jars, at 3 × 10^6^ cells per jar (surface area approx. 15 cm^2^). These cultures were supplemented every other day by addition of 0.75 mL fresh HMDM medium, and medium was exchanged 7 days after initial seeding.

Adherent cell lines were maintained in DMEM complete medium (DMEM/10) containing 1 mg/mL L-glutamine, 1 mM sodium pyruvate, 10 mM HEPES, 1X Pen/Strep, and 10% fetal bovine serum. Lymphoid cells (Molt4-CCR5) were propagated in RPMI containing 1 mg/mL L-glutamine, 1 mM sodium pyruvate, 10 mM HEPES, 1X Pen/Strep, and 10% fetal bovine serum.

### Viruses

HIV-1 and lentiviral molecular clones were gifts or obtained through the NIH AIDS Reagent Program (ARP), Division of AIDS, NIAID, NIH: WT-ADA and R9-∆ENV (from Mario Stevenson, U.Miami CFAR); pNL43-(BaL env)-nef-IRES-GFP (from Thorsten Mempel, MGH, and Thomas Murooka, U.Manitoba); pWT-BaL (from Bryan R. Cullen, Duke Medical Center via ARP, Cat#11,414); pNL-GFP-RRE(SA) (from Dr. Jon Marsh and Dr. Yuntao Wu, NIH/NIMH via NIH ARP Cat# 11,466). HIV-nef-IRES-mCherry was generated by cloning the mCherry cDNA into HIV-nef-IRES-GFP, using PCR amplification from pCAAGS-mCherry (Natasza Kurpios, Cornell University) and cloning a 1.7 kb MluI-IRES-mCherry-LTR-XbaI cassette into digested pNL43-(BaL env)-nef-IRES-GFP.

### Production of virus stocks

Infectious HIV molecular clones or lentiviral vector pNL-GFP-RRE(SA) were transfected into 293FT cells with or without pLP-VSV-G (Invitrogen) and pCMV-dR8.2-dvpr (Bob Weinberg, Addgene #8455)^12^. Transfection was performed in T-150 culture flasks using Lipofectamine 2000. Medium was replaced after overnight incubation with fresh complete medium containing Pen/Strep, and cultured for one additional day. Supernatants were harvested on days three and four, pooled, cleared by centrifugation, filtered through a 0.45 μM PVDF ultra low protein-binding filter (Millipore) and aliquoted for storage at − 80 °C. Viral titer was established on single freeze–thaw cycle stocks by limiting dilution and TCID50/ml was calculated using the Reed-Muench method^13^.

### Infections

Inoculations of HMDM were performed in Teflon screwtop 60 mL jars (Savillex) in 2 mL of fresh medium without added DEAE-dextran or polybrene. Short-duration macrophage infections with VSV-G enveloped virus (single round R9Δ*env* and double-enveloped VSV-G-BaL) were performed on 5 × 10^5^ HMDM with an MOI of 1. Inoculum medium was changed after 24–48 h, and cells were incubated in complete macrophage medium for an additional 2–14 days for viral replication. TZM-gfp cells (2.5 × 10^5^) were seeded in duplicate in 35 mm glass-bottom plates (ibidi) in the morning, allowed to adhere, and infected 6–8 h later in 1.5 mL total fresh medium with a panel of HIV-1 stocks (Fig. 1C–N) at MOI 0.1. Inocula were exchanged for 2 mL fresh medium the following morning, and incubated an additional 24–48 h before analyses by flow cytometry or live confocal imaging.

### Comparative titration assay

TZM-gfp and TZM-bl cells (8 × 10^4^) were infected in triplicate with a fivefold titration series of HIV-1 BaL beginning at MOI 0.5 in 24-well dishes. At 48 h post-infection, wells for flow cytometry were washed and trypsin-harvested, fixed for 30 min in Cytofix/Cytoperm (BD), then stained with anti-p24 (KC57:RD1). Entire cultures from each well and dilution were run on a BioRad S3e instrument acquiring p24-RD1 and GFP intensity, and the results were analyzed in FlowJo 10 (BD). Single cells were gated on GFP and p24 as indicated in Fig. 6, and population statistics were plotted using Prism 6 (GraphPad). Replicate cultures of TZM-bl at 48 h were lysed using the Firefly Luc One-Step Glow Assay Kit (Pierce) per manufacturer instructions in 400 uL total. After 10 min, lysate was homogenized by pipetting, and 100uL were transferred to opaque white 96-well assay plates (Corning) for luminescence analysis on an Envision plate reader instrument (Perkin Elmer).

### Co-culture cell density titrations

HMDMs (5 × 10^5^) were infected with HIV-1 ADA (0.7 MOI) in PTFE 60 mL screwtop jars (Savillex). Inoculum media was replaced the following day with 5 mL fresh HMDM medium, and cells were incubated for 6 more days, supplementing 0.75 mL fresh HMDM medium every other day. On the day of co-culture, cells were harvested by gentle pipetting, counted with a hemocytometer, and 100 viable cells were added to pre-established TZM-gfp monolayer cultures of varying densities (5 × 10^3^–1 × 10^5^ TZM-gfp cells/cm^2^) on 35 mm glass-bottom plates (ibidi). Live cultures were imaged every 24 h for five days post-infection using a Leica SP5 scanning confocal microscope under BSL3 containment.

### Drop culture

TZM-gfp cells were trypsin-harvested and resuspended at ~ 10^7^/mL in a sterile microfuge tube. A series of 20 twofold dilutions was prepared, with dilutions down to 20 cells/mL (0.04 cells per 2 μL). From each dilution tube and starting from the most dilute, 2 μL were spotted onto 35 mm glass-bottom plates (ibidi) in a 5 × 5 spot array. Eight spots of 20uL each were spotted around the outer rim of culture plastic to provide humidification within each plate. Plates were sealed with parafilm and incubated overnight for cell adherence within each drop. The following morning, the plate was washed with fresh medium and inoculated with HIV-1 ADA supernatant, such that all foci shared the same viral inoculum of 1 × 10^5^ TCID50 doses in 1.5 mL total for each plate. Plates were washed 24 h later, and incubated for two additional days before being analyzed for reporter signal by live confocal microscopy under BSL3 containment.

### Primary HIV-1 capture from clinical samples in Blantyre, Malawi

Twenty-four hours prior to co-culture, 200,000 TZM-gfp or Molt4-CCR5 cells were plated in 6-well dishes on coverslips. PBMCs were obtained from an HIV-infected ART-naïve Malawian adult individual by venipuncture and prepared by Lymphoprep (Axis-Shield) per manufacturer instructions. PBMC (250,000) were added to TZM-gfp or Molt4-CCR5 coverslip cultures. At 24 or 48 h following co-culture, supernatants were harvested and cleared by centrifugation, then stored at − 80 °C. Coverslips were submerged in fixative containing 2.5% glutaraldehyde in 0.1 M sodium cacodylate buffer, pH 7.2 in a Wash-N-Dry coverslip rack (Diversified Biotech), and sealed in a teflon jar (Savillex) for storage and transport at room temperature.

### Secondary HIV-1 outgrowth

Molt4-CCR5 cells (1 × 10^4^) were spinoculated with 500uL primary, untitered co-culture supernatants for 60 min at 1000×*g* in the presence of 20 μg/mL DEAE-dextran in 1.5 mL total. Cells were incubated in 5 mL for 3 days, when an additional 3 mL fresh medium was added. Cultures were supplemented in this way with increasing volumes of fresh medium (3–10 mL) every 3–4 days. On Day 13, the culture was split and 1 × 10^6^ fresh, uninfected and DEAE-dextran-treated Molt4-CCR5 cells were added to a twofold excess of cells from the infected outgrowth culture and spinoculated as described above. Cultures were then resuspended in 10 mL total, incubated for 3 days, then supplemented with 20 mL of fresh medium for a final 4-day incubation. Infectious viral supernatant was harvested on day 21, cleared by centrifugation, 0.45 μm PVDF-filtered, and aliquoted for storage at − 80 °C.

### Electron microscopy

Thermanox coverslips were rinsed 3× for 10 min in sodium cacodylate buffer, then fixed for 1 h in buffered 2% osmium tetroxide and stained in 1% aqueous uranyl acetate. Coverslips were rinsed in H_2_0, then dehydrated in an ethanol series. The samples were infiltrated with UltraBed Epoxy resin through a resin:ethanol series. Infiltrated coverslips were inverted over the end of a Beem capsule filled with resin and polymerized overnight. The Thermanox coverslips were snapped off under liquid nitrogen, trimmed and sectioned. Grids were stained in uranyl acetate and lead citrate and imaged on an FEI Tecnai 12 Bio-twin transmission electron microscope.

### P24 ELISA

Supernatant samples were applied to the HIV-1 Type 1 p24 Antigen ELISA (Zeptometrix) and the ELISA was performed using a dilution series of known standards of purified p24 protein, according to the manufacturer instructions.

### Flow cytometry

Macrophages were harvested for flow cytometry from Savillex PTFE jars by gentle pipetting. TZM-gfp cultures (including co-cultures) were washed with cold PBS, then trypsin-harvested with gentle pipetting. Cells were pelleted by centrifugation at 200xg, washed twice in FACS buffer (0.1% BSA in PBS), then resuspended in Cytofix/Cytoperm (BD) for 1 h at room temperature. Cells were again washed with FASC buffer, filtered through a 70 µM nylon strainer (Fisher), and stained with conjugated antibodies in 100uL final volume in the dark. Stained samples were washed and run on a BD LSRII instrument. Data were analyzed using FlowJo software. Antibodies for flow cytometry included: Anti-p24-RD1 (KC57, Beckman Coulter), Anti-CD14-PE (M5E2, BD), Anti-CCR5-PE (2D7, BD), Anti-CD4-APC (RPA-T4, BioLegend), Anti-CXCR4-APC (12G5, BioLegend).

### Protein transfections

TZM-gfp cells (15,000 per well) were seeded in 8-well chamber glass slides (ibidi) in DMEM/10 mendium and allowed to adhere overnight. The following morning the medium was replaced with 200ul of fresh DMEM10 without antibiotics. Purified protein mixtures (300 ng Rev+/− 300 ng Tat) were then transfected using 0.6uL of CrisprMAX reagent (Invitrogen) without Cas9 Plus Reagent, per manufacturer instructions. Cells were incubated 6 h, washed three times with fresh medium, then fed with 200uL fresh DMEM10 medium without antibiotics. Extracellular Tat was then added at 300 ng per well in 200uL total medium and incubated for 24–48 h. Live cells were analyzed by confocal microscopy for induction of GFP reporter signal using a Leica SP5 instrument.

### Human subjects

All study participants recruited in Malawi gave written informed consent. The study was approved in Malawi by the College of Medicine Research Ethics Committee (COMREC; protocol P.05/15/1728) and in the United Kingdom by the Liverpool School of Tropical Medicine Research Ethics Committee (LSTM REC; protocol 15.026). All procedures were conducted in accordance with the relevant guidelines and regulations.

### Consent for publication

All authors have read the manuscript and consent to its publication and information about themselves as authors. Signed consent declarations available upon request.

## Data Availability

All data generated or analyzed during this study are included in this published article; any data not shown is available upon request of the authors. TZM-gfp cells will be made available through the NIH AIDS Reagent Program upon publication of this work.
